# Glycopeptoid nanospheres: glycosylation-induced coacervation of poly(sarcosine)†

**DOI:** 10.1039/d2na00218c

**Published:** 2022-06-27

**Authors:** Yota Okuno, Tomoki Nishimura, Yoshihiro Sasaki, Kazunari Akiyoshi

**Affiliations:** Department of Polymer Chemistry, Graduate School of Engineering, Kyoto University Kyoto Daigaku-Katsura, Nishikyo-ku Kyoto 615-8510 Japan akiyoshi.kazunari.2e@kyoto-u.ac.jp; Department of Chemistry and Materials Engineering, Faculty of Chemistry, Materials and Bioengineering, Kansai University, 3-3-35, Yamanote-cho Suita City Osaka Japan; Department of Chemistry and Materials, Faculty of Textile Science and Technology, Shinshu University, 3-15-1 Tokida Nagano 386-8567 Japan

## Abstract

Conjugation of maltopentaose to water-soluble homo-poly(sarcosine) induced self-association and formed nanospheres (−150 nm) in water although homo-poly(sarcosine) was water-soluble and did not form any aggregates. Fluorescent probe experiments showed that the spheres were non-ionic glycopeptoid coacervate-like particles with both hydrophobic and hydrophilic domains inside.

## Abstract 2

Conjugation of maltopentaose to water-soluble homo-poly(sarcosine) induced self-association and formed nanospheres (−150 nm) in water although homo-poly(sarcosine) was water-soluble and did not form any aggregates.

## Graphical abstract

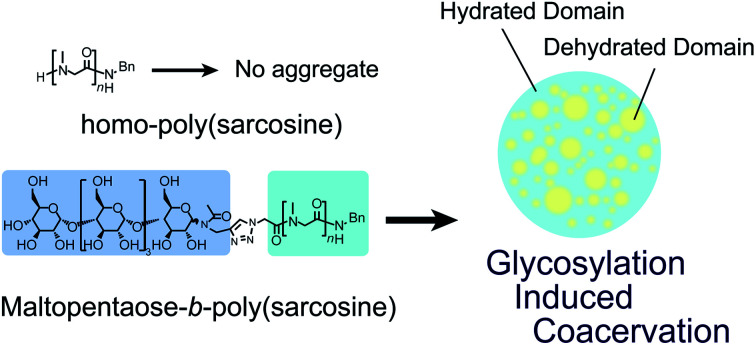

Recently there has been great interest in coacervates, which form membraneless organelles containing proteins and RNA^1,2^ in living systems.^1–6^ In general, a coacervate is one of the self-associations of the macromolecule and the macromolecule-rich phase does not form a specific structure (for example, a vesicle, micelle, or tube). Researchers have investigated coacervates not only in terms of fundamental polymer science but also for biomedical applications, such as drug delivery systems^7–10^ and an enzymatic reaction field.^6,11,12^

Polyelectrolytes—which are formed mainly by ionic interactions among polymer chains—are well-studied because of their physicochemical properties,^13–15^ such as phase formation^16–18^ and the size distribution of the droplets.^19^ In contrast to polyelectrolyte coacervates, there are a few reports on nonionic polymer coacervates. Nonionic elastin-analogues having repeat VPGXG-motifs exhibit temperature dependent coacervation *via* hydrophobic interactions and hydrogen bonding among amino acids.^20–22^ Polymers inspired by elastin or tropoelastin motifs were designed and also formed coacervates.^23,24^

Regarding the polymer colloidal science related to coacervates, double hydrophilic block copolymers [such as dextran-*b*-poly(ethylene oxide), dextran-*b*-poly(sarcosine), and pullulan-*b*-poly(ethylene oxide)] form by self-assembly in water and can also form micrometer-sized giant vesicles in water.^25–27^ Pullulan-*b*-poly(acryl amide) and pullulan-*b*-poly(vinyl pyrrolidone) also form nano-sized spheres in water. Researchers explain the self-association of such double hydrophilic block copolymers based on polymer–polymer phase separation which is related to well-known two-phase polymer systems, such as polysaccharides and polyethylene glycol.

We report here a new non-ionic polymer based coacervate-like nanosphere consisting of a glycopeptoid, maltopentaose conjugated poly(sarcosine) (Fig. 1). Poly(sarcosine) is a water-soluble, non-ionic peptide derivative and is suitable for biomedical applications because it is biocompatible, biodegradable and stealth to our immune system.^28–32^ Additionally, monodisperse and narrow molecular weight distribution poly(sarcosine) was obtained *via N*-carboxyanhydride (NCA) polymerization. We previously reported that carbohydrate-conjugated thermoresponsive peptoid (poly(*N-n*-propylglycine) self-associated in water above LCST and formed a vesicular structure.^33^ In this study, conjugation of maltopentaose (only five sugar monomers) to water-soluble homo-poly(sarcosine) induced self-association, coacervation and enabled the formation of monodisperse nanospheres in water, although homo-poly(sarcosine) did not form any aggregates. The glycopeptoid nanosphere is interesting for potential application as a nanocarrier for drug delivery systems. The phenomenon reported here is also pertinent to glycosylation-induced coacervation of water-soluble peptoids.

**Fig. 1:**
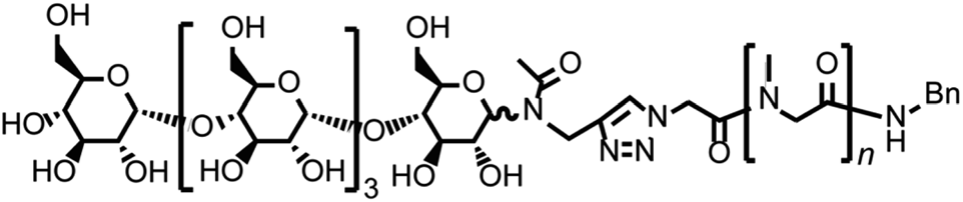
The chemical structure of the glycopeptoid (compound [2]).

Schemes S1–S3† show the synthetic schemes for conjugating maltopentaose to poly(sarcosine). We first synthesized the peptoid segment by polymerization of sarcosine NCA.^34^ Benzylamine was injected as an initiator into 1 M sarcosine NCA solution in *N*,*N*-dimethylformamide (DMF) in which [benzylamine] : [NCA] = 10 : 1000 on a millimole-to-millimole basis, and the solution was stirred under an argon atmosphere at 20 °C for 12 h. The size-exclusion chromatogram of the resulting polymer (compound [1]) indicated a single peak with a very narrow molecular weight distribution (*Đ*_M_ = 1.04; Fig. S1†). The molecular structure was confirmed by ^1^H-NMR spectroscopy in MeOH-*d*_4_ (Fig. S2†). In the second step, we used azidoacetic acid as a capping agent for poly(sarcosine) at the *N*-terminus. We also synthesized alkyne-functionalized maltopentaose asthe glyco-segment (Fig. S3†).^33,35^ Finally, alkyne-functionalized maltopentaose was conjugated to azide-functionalized poly(sarcosine) by copper-catalyzed azide-alkyne cycloaddition (Scheme S3†). The resulting glycopeptoid (compound [2], Fig. 1), maltopentaose conjugated poly(sarcosine), also exhibited a narrow molecular weight distribution (*Đ*_M_ = 1.03; Fig. S1†). Additionally, the retention time of the size exclusion-chromatography peak shifted to the high molecular weight region, suggesting that the carbohydrate group was introduced into the homo-poly(sarcosine). The degree of poly(sarcosine) units in the glycopeptoid was confirmed by ^1^H-NMR spectroscopy in MeOH-*d*_4_ to be 86 (Fig. S4†). The MALDI-TOF-MS data of the glycopeptoid showed a peak at 7232.59 *m*/*z*, corresponding to the calculated mass 7232.59 *m*/*z* of maltopentaose-conjugated poly(sarcosine)_86_ with a sodium ion (Fig. S5†).

The solvent of the methanol solution of the glycopeptoid [maltopentaose conjugated poly(sarcosine)_86_] was removed under reduced pressure to obtain the thin polymer film. Then water was added to the resulting film for a polymer concentration of 10.0 mg mL^−1^. The film immediately dissolved and a transparent solution was obtained. The solution of the glycopeptoid (compound [2]) was sonicated for 1, 5, and 10 min with a bath type sonicator at 20 °C (40 kHz, 130 W). The particle sizes of the *Z*-averages determined by dynamic light scattering (DLS) were 149 nm [polydispersity index (PDI) = 0.196] after 1 min, 150 nm after 5 min (PDI = 0.181), and 150 nm after 10 min (PDI = 0.192) (Table S1†). The glycopeptoid formed monodisperse nanospheres. On the other hand, the size of homo-poly(sarcosine) (compound [1]) solution was 2.3 nm (PDI = 0.494) and was probably dispersed as monomer poly(sarcosine) (Fig. 2a). It is noteworthy that the terminal conjugation of hydrophilic poly(sarcosine) to hydrophilic maltopentaose induced changes in solution properties drastically. When the concentrations of the glycopeptoid solution (1.0–10.0 mg mL^−1^) were decreased, the size of the nanospheres did not change so much though the sizes slightly decreased with decreasing concentration (Table S2, Fig. S6†). Transmission electron microscopy (TEM) of the glycopeptoid solution showed spherical structures and the diameters between 50 and 150 nm (Fig. 2b). The magnified TEM and cryo-TEM images have homogeneous contrast in the interior of the sphere; a coacervate (Fig. 2c and d).

**Fig. 2:**
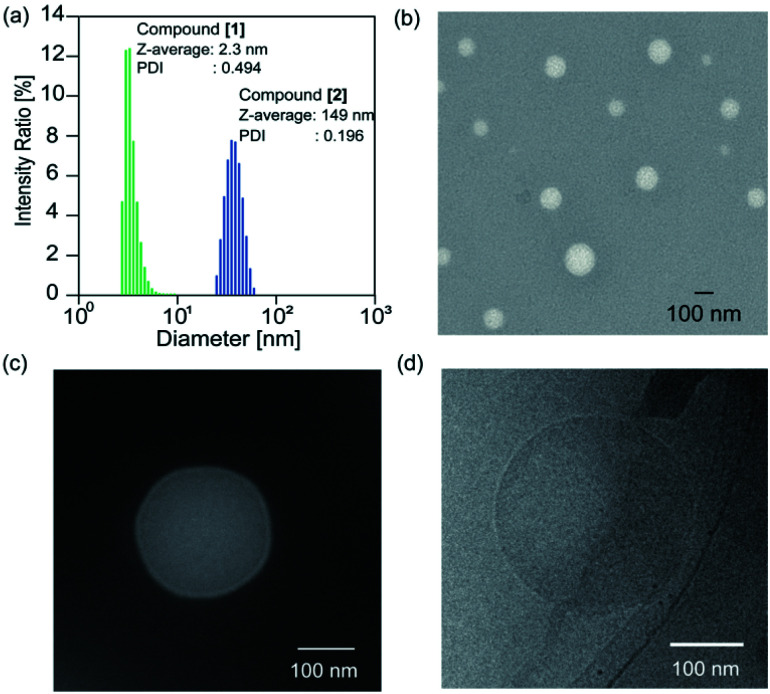
(a) Size distribution of self-assembly of maltopentaose-conjugated poly(sarcosine)_86_ (compound [2]) and homo-poly(sarcosine) (compound [1]) in water, determined by DLS. (b) TEM image, with negative staining using ammonium molybdenum, of a self-assembled maltopentaose-conjugated poly(sarcosine)_86_ in water (10.0 mg mL^−1^). (c) Magnified TEM image of an individual vesicle (10.0 mg mL^−1^). (d) Cryo-TEM image of self-assembled maltopentaose-*b*-poly(sarcosine)_86_ in water (10.0 mg mL^−1^).

To evaluate the internal microenvironment of the nanospheres, we carried out fluorescence measurements using pyrene as a hydrophobic probe (Fig. 3a). The intensity ratio between the first and third peak of the pyrene fluorescence emission spectrum is related to the micro polarity (*e.g.*, hydrophobicity) around a pyrene molecule; the higher *I*_1_/*I*_3_ ratio reflects the environment with more hydrophilicity. When we applied homo-poly(sarcosine) (compound [1]) in water at 30 °C (10 mg mL^−1^), the peak of the pyrene dimer was evident and the *I*_1_/*I*_3_ ratio as a parameter of the polarity was 1.61, which suggests a quite hydrophilic environment (1.87 in water).^36^ However, in the presence of glycopeptoid (compound [2]) solution at 20 °C (10.0 mg mL^−1^), no dimer peak was evident and the *I*_1_/*I*_3_ ratio decreased to 1.46 (same as a cyclopentanone).^36^ The sizes and *I*_1_/*I*_3_ ratios of the nanospheres did not change so much depending on the temperature (from 10 °C to 40 °C) (Table S3, Fig. S7–S9†). Pyrene was trapped in the glycopeptoid sphere and the microenvironment was relatively hydrophobic compared to the homo-poly(sarcosine) solution.

**Fig. 3:**
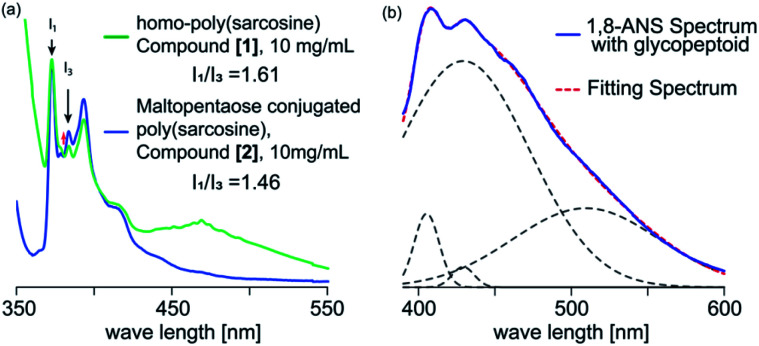
(a) Fluorescence emission spectra of pyrene in homo-poly(sarcosine) and maltopentaose conjugated poly(sarcosine) solution at 20 °C (10.0 mg mL^−1^). The solution was excited at 339 nm. (b) Fluorescence emission spectrum of 1,8-ANS solution (20 mM) in the presence of glycopeptoid recorded at 20 °C (10.0 mg mL^−1^). The dispersions were excited at 350 nm. The obtained fluorescence spectrum was fitted by Gaussian curves. The solid blue line: measured 1,8-ANS spectrum, grey dotted line: deconvoluted line and red dotted line: the sum of grey lines.

For further investigation into the microenvironment, 1-anilinonaphthalene-8-sulfonic acid (1,8-ANS) was used as a second probe, which is a solvatochromic fluorescence probe so that the emission spectrum of 1,8-ANS depending on the surrounding environment polarity i.e. hydrophobicity.^37–39^ Fluorescence at a wavelength beyond 500 nm was observed in polar environments, while that below 470 nm was in non-polar environments. For example, the intense emission peak top wave lengths in water, dioxane, ethanol and *n*-heptane are observed at 535, 471, 464 and 400 nm, respectively.^37–41^ The quantum yield of 1,8-ANS in water (broad peak of 535 nm) is quite low due to hydration. Even in the presence of homo-poly(sarcosine), the fluorescence emission did not change at all (broad peak of 535 nm). However, in the presence of glycopeptoid, strong emissions below 470 nm were observed (200 times higher than that in the presence of homo-poly(sarcosine) (Fig. 3b). The spectra were deconvoluted with gaussian curves involving the peak of the species with various polarities. The deconvoluted data were shown by the peak top wave length corresponding to the microenvironment of at least three micropolarities (two hydrophobic environments: 400 and 410–420 probably exist inside the glycopeptoid sphere, and a hydrophilic environment: 510). These data suggest that the conjugation of maltopentaose to poly(sarcosine) partly accelerated dehydration of the peptoid and induced self-association (*i.e.* coacervation) with various domains inside.

Giant spheres were observed by just hydration of the film of the samples without sonication. To obtain further information about the structure of the sphere, confocal laser scanning microscopic measurement of the giant spheres was carried out. The film of the glycopeptoid was dissolved in water (a polymer concentration of 10.0 mg mL^−1^) and fluorescein (green) and/or rhodamine 6G (red) as a hydrophilic or hydrophobic fluorescent probe, respectively, were added to the solution without sonication. The spheres with average diameters within the range of 3–5 μm were observed by confocal laser scanning microscopy (Fig. 4). Both probes were trapped in the sphere and emitted fluorescence from the interior of the sphere. The images suggest that the glycopeptoid self-associated and formed spheres with internal domains consisting of both hydrophobic and hydrophilic microenvironments, which are typically shown in the coacervate. These data indicate that the glycopeptoid formed coacervate-like spheres in water.

**Fig. 4:**
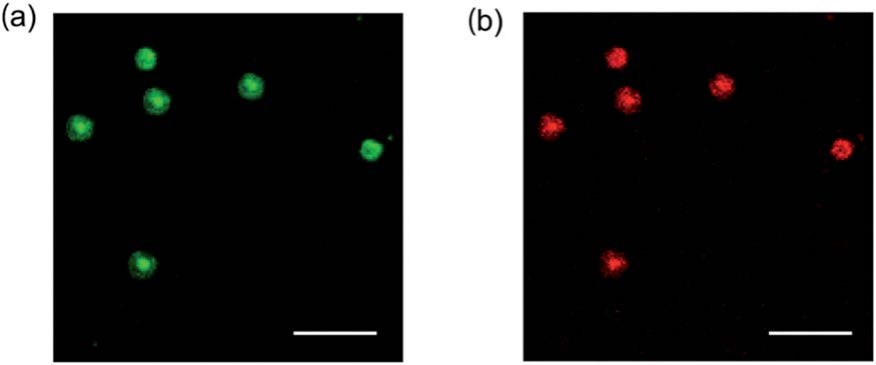
Confocal laser scanning microscopy images of glycopeptoid in water (without sonication). Fluorescence emissions from (a) fluorescein and (b) rhodamine 6G; all scale bars: 10 μm.

The effects of NaCl and urea (Table S4, Fig. S10 and S11†) were investigated. To the thin films of the glycopeptoid in a glass tube, NaCl or urea solutions of various concentrations (final polymer concentrations, 10.0 mg mL^−1^) were added and the solution was sonicated for 1 min with a bath-type sonicator. The sizes of the spheres did not significantly change with increasing NaCl concentration, although in general polyelectrolyte coacervates collapse in high-salt concentrations.^42^ The morphology in 4.0 M aqueous NaCl was checked by TEM and similar spherical structures were observed in comparison with those in water (Fig. S12†). However, the sizes and sphere count rate in DLS decreased with increasing urea concentration (Table S4†). The TEM images of self-assemblies in 8 M aqueous urea indicated smaller nanospheres than those in water (Fig. S13†). The nanospheres probably collapsed in the presence of urea, a hydrogen bond inhibitor. These results suggest that hydrogen bonding among maltopentaose or poly(sarcosine) chains affected the formation and stability of the self-association.

Because the nanospheres collapsed especially in the presence of urea as a hydrogen bond inhibitor, one of the driving forces of the association (coacervation) of the glycopeptoid could be a hydrogen bonding interaction. There are no donor molecules for hydrogen bonding (hydrogen molecules) and only acceptor molecules (carbonyl groups) in poly(sarcosine). However, in carbohydrate-conjugated poly(sarcosine), the hydrogen bonding between the OH groups of the oligosaccharide and the carbonyl groups of the poly(sarcosine) chain probably induces intra-macromolecular or inter-macromolecular association of the glycopeptoids. In addition, the association by the hydrogen bonding with the oligosaccharides can induce the dehydration of the adjacent poly(sarcosine) chains. As a result, the hydrophobic domains by the association of partly dehydrated poly(sarcosine) chains are formed. Finally, we proposed that carbohydrate-conjugated poly(sarcosine) self-associated by both hydrogen bonding and hydrophobic interaction, and formed nano- or micro-spheres (coacervates) with hydrophilic and hydrophobic microdomains without specific structures (Fig. S11†).

## Conclusions

We characterized the self-assembly of a non-ionic glycopeptoid, maltopentaose conjugated poly(sarcosine)_86_, in this study. The glycopeptide formed monodisperse coacervate-like nanospheres. This is a (to the best of our knowledge) new finding: facilitating association of water-soluble poly(sarcosine) by introducing only a small number of sugar monomers into the poly(sarcosine) terminus. These non-ionic nanospheres can be used as a new type of nanocarrier because the particles were stable even in high concentrations of NaCl; polyelectrolyte coacervates may not be stable under physiological conditions. This glycoconjugate hydrophilic polymer will facilitate the design of further polymer-forming, non-ionic polymer coacervates. This glycosylation-induced coacervation phenomenon is also pertinent to testing hypotheses of modulating coacervation in membraneless organelles.^43^

## Conflicts of interest

There are no conflicts to declare.

## Supplementary Material

NA-004-D2NA00218C-s001
