# Supplementary material for: Glycopeptoid nanospheres: glycosylation-induced coacervation of poly(sarcosine)
Source: Nanoscale Adv. 2022 Jun 27;4(18):3707–10. doi: 10.1039/d2na00218c (PMC9470024; doi:10.1039/d2na00218c)
Supplement: NA-004-D2NA00218C-s001 [file NA-004-D2NA00218C-s001.pdf]

Electronic Supplementary Material (ESI) for Nanoscale Advances.
This journal is © The Royal Society of Chemistry 2022

Supporting information

for

Glycopeptoid Nanospheres: Glycosylation-Induced Coacervation of Poly(sarcosine)

Yota Okuno[1], Tomoki Nishimura[2]*, Yoshihiro Sasaki[1], and Kazunari Akiyoshi[1]*

1. Department of Polymer Chemistry, Graduate School of Engineering, Kyoto University, Katsura, Nishikyo-ku, Kyoto 615-8510, Japan.

2. Department of Chemistry and Materials, Faculty of Textile Science and Technology, Shinshu University, 3-15-1, Tokida, Nagano 386-8567, Japan

Experiment

Materials.

Maltopentaose (purity > 98%), acetic anhydride (purity > 93%), ammonium molybdenum (purity > 99.98%) and copper(I) bromide (purity > 95%) were purchased from Wako Pure Chemical Industries, Ltd, Japan. Propargyl amine (purity > 95%), sarcosine (purity > 98%), methyl chloroformate (purity > 96%), thionyl chloride (purity > 98%), benzylamine (purity > 99%), azidoacetic acid (purity > 97%), pyrene (purity > 97%) and fluorescein isothiocyanate (purity > 97%) were purchased from Tokyo Chemical Industry Co., Ltd, Japan. Rhodamine B and fluorescein were purchased from Sigma–Aldrich Co. LLC, USA.

DLS Measurement. DLS was carried out with a Zetasizer Nano ZS instrument (Malvern Instruments, U.K.) operating at a wavelength of 632.8 nm (10 mW) and a 173° detection angle at 20 °C.

Fluorescence measurements: Fluorescence spectra were recorded with a fluorescence spectrophotometer (FP-8500, JASCO, Japan). Solutions consisting of either polymer in methanol or a stock solution of pyrene ([pyrene] = 1.0×10^{-4} M) in ethanol were added to a vial. Then the solvent was evaporated to afford the polymer thin film. Pure water was added to the polymer film and the solution was sonicated at 20 °C for 1 min. The final concentrations of the polymer and pyrene in the solution were 10.0 mg mL^{-1} and 1×10^{-6} M, respectively. Pyrene was excited at 339 nm. The slit width was set to 5 nm for the excitation and 5 nm for the emission with highly sensitive mode. Fluorescent measurements were carried out at 10 °C, 20 °C, 30 °C, 40 °C.

Transmission electron microscopy: Five microliters of the polymer solutions (1.0 mg mL^{-1}) in pure water were placed on a copper grid coated with an elastic carbon film (ELS-C10 STEM Cu100P, Okenshoji Co., Ltd. Japan). Excess sample was removed with filter paper. Five micro-liters of 2 wt% ammonium molybdian acid solution as a staining agent was added and then removed. The sample was dried in a desiccator. The grid was placed in a HT-7700 (Hitachi, Tokyo, Japan) electron microscope operated at 100 kV.

CLSM observations of giant self-assembly: A methanol solution of the block polymer was added into a vial and the solvent was evaporated under reduced pressure. The obtained thin polymer film was hydrated with pure water. Then a fluorescent dye (rhodamine 6G or fluorescein) was added to the giant self-assembly solution. The images of giant vesicles were acquired by CLSM (LSM780; Carl Zeiss, Germany) at a magnification of 63× with an excitation wavelength of 488 nm.

Cryo Transmission electron microscopy: Five micro-liters of the polymer solutions (10.0 mg mL^{-1}) in pure water were placed on a copper grid coated with an elastic carbon film (NS-C075 STEM Cu75P,

Okenshoji Co., Ltd. Japan). Excess sample was removed with filter paper. The sample on the grid was instantly freezed by some liquid propane (-196 °C) with Reichert KF80 (Leica, Japan). The freezed grid was placed in a JEM-2100F(G5) (JEOL, Japan) transmittance electron microscope operated at 200kV.

Synthesis

All starting materials and solvents were used without further purification (**Scheme S1**). Nuclear magnetic resonance spectra were run in methanol-d_4 with a Bruker Avance III 400MHz spectrometer to acquire ^{1}H-NMR spectra. Chemical shifts (δ) are expressed in parts per million and are reported relative to the solvent peak as an internal standard in ^{1}H-NMR spectra. Mass spectra were recorded with a Thermo Exactive spectrometer. The molecular weight distribution (MWD) curves, number-average molecular weights (M_n), and polydispersity indices ($Đ_M$) were measured by SEC in DMF containing 10 mM LiBr at 40 °C (flow rate = 0.50 mL min^{-1}) with three linear-type polystyrene gel columns (TOSOH TSKgel SuperHM-M; exclusion limit: 4 × 10^6 g mol^{-1}; particle size: 3 µm; pore size: N/A; 6.0 mm i.d. × 15 cm) and an HLC-8320GPC (TOSOH) instrument equipped with refractive index and UV–visible detectors ([illegible] = 254 nm). The columns were calibrated against 12 poly(methyl methacrylate) standards [PSS: M_p=8.00×10^2 to 2.20×10^6 g mol^{-1}; $Đ_M$(SEC) = 1.04–1.22].

Scheme S1

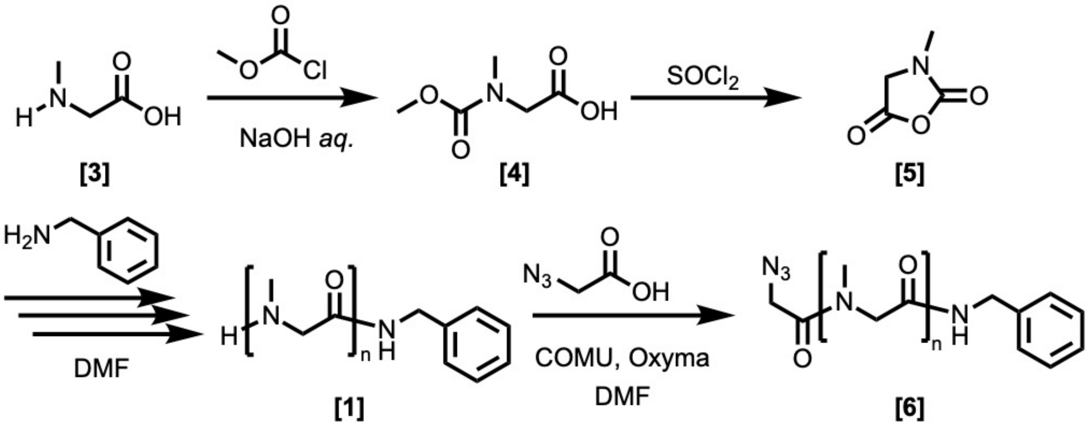

Compound [4]: *N*-(methoxycarbonyl)-*N*-methylglycine

Sarcosine (30.0 g, 337 mmol) was dissolved in 337 mL of 1 M aqueous NaOH and methyl chloroformate (33.9 mL, 438 mmol) was dropwised under cooling with a 0 ºC ice bath. The mixture was stirred for 6 h at room temperature. Then the pH of the solution was adjusted from 11 to 6 with 2 N aqueous HCl. The compound was extracted by diethyl ether. The solvent was removed under reduced pressure.

Compound [**4**] was a white solid (42.54 g, 288 mmol, 84%). ^{1}H NMR (400 MHz, methanol-d_4): δ= 2.94, 2.95 (*d*, *J*=4Hz, 3H, CH_3OCO), 3.67, 3.71 (*d*, *J*=16Hz, 3H, NCH_3(CH_2)COOH), 3.98, 3.99 (*d*, *J*=4Hz, 2H, NCH_3(CH_2)COOH)

Compound [5]: sarcosine-*N*-carboxyanhydride (NCA)

Compound [**4**] (5.00 g, 34.0 mmol) was dissolved in $SOCl_2$ (50 mL, 0.693 mol) and the solution was stirred for 12 min in an oil bath at 70 °C. The solution was reprecipitated by pouring into petroleum ether. The crystals were gathered by filtration and dried *in vacuo*. The dried crystals were dissolved into dry ethyl acetate at 50 °C and the solution was passed *via* activated carbon. The solvent was removed under reduced pressure at room temperature.

Compound [**6**] was a colorless oil (1.28 g, 11.12 mmol, 33%).

Polymer [1]: Poly(sarcosine) homopolymer

Compound [**5**] (1.16 g, 10.1 mmol) was dissolved in dry dimethylformamide (10 mL) and benzylamine (10.8 µL, 101 µmol) was injected into the solution. The mixture was stirred for 12 h at room temperature under an argon atmosphere. Then the polymer solution was added to diethyl ether and reprecipitated.

The obtained solid was a white powder (550 mg, 76%). $Đ_M$(SEC)=1.04. ^{1}H NMR (400 MHz, methanol-d_4): δ=2.93–3.06 (*broad*, 249H, NCH_3), 4.10–4.52 (*broad*, 166H, N(CH_3)CH_2CO), 7.29–7.33 (*q*, *J*=8 Hz, 5H, *aromatic*) [Figure S1].

Compound [7]: Azido-functionalized poly(sarcosine) homopolymer

Compound [**1**] (100 mg, 13.9 µmol) was dissolved in 3 mL of dry DMF. COMU (25.2 mg, 58.9 µmol), Oxyma (8.39 mg, 58.9 µmol), and 2-azidoacetic acid (10 µL, 134 µmol) were added to the solution, followed by addition of *N*,*N*-diisopropylethylamine (10 µL, 101 µmol). The mixture was stirred for 24 h at room temperature. The solvent was removed under reduced pressure and the residue was washed with ethyl acetate.

The obtained solid was a slightly yellow powder (90 mg, 12.3 µmol, 88%).

Scheme S2

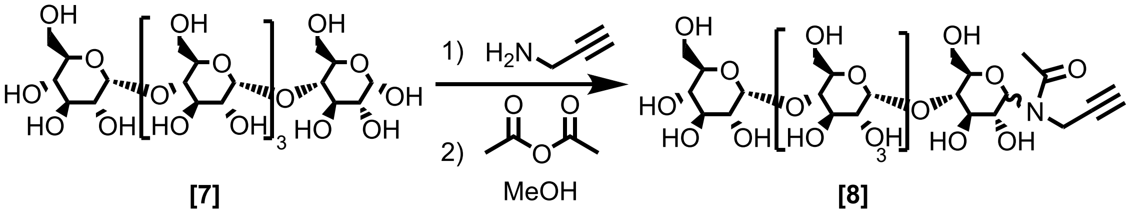

[7] [8]

Compound [8]: Alkyne-functionalized maltopentaose

Maltopentaose (compound [**7**], 1.09 g, 1.31 mmol) was dissolved in propargylamine (2.6 mL, 40.6 mmol) and the mixture was stirred for 144 h at room temperature. Then the solution was added dropwise to diethyl ether and the resultant white solid was separated by filtration. The white solid was dissolved in the mixture of dry methanol (280 mL) and acetic anhydride (15.0 mL, 159 mmol). The solution was stirred for 144 h at room temperature. The solvent was removed under reduced pressure and the residue was dried *in vacuo*.

The obtained solid was a white powder (1.06 g, 89%). ^{1}H NMR (400 MHz, methanol-d_4): δ= 2.22 and 2.28 (2 × *s*, 3H, rotamers, NCOCH_3), 2.49 and 2.77 (2 × *s*, 1H, rotamers, C≡C*H*), 3.4–4.16 (*br*, 46H, sugar backbone), 5.15–5.20 (*m*, 4H, anomeric protons) 4.91and 5.53 (*d*, *J*=8 Hz, 1H, anomeric proton) [Figure S2].

Scheme S3

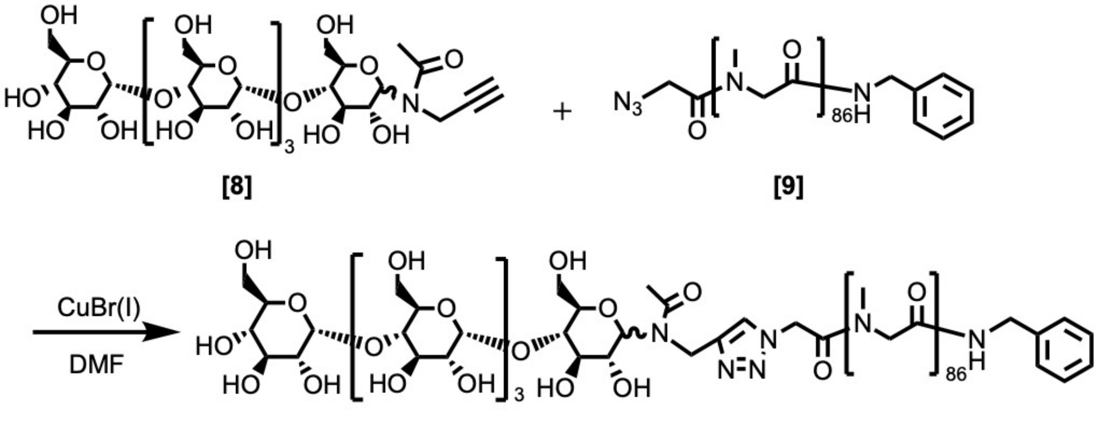

Polymer [2]: Maltotriose-*b*-poly(*N*-*n*-propylglycine)

Compound [**8**] (40.0 mg, 44.1 µmol) and compound [**9**] (50.0 mg, 7.93 µmol) were dissolved in 2 mL of dry DMF. CuBr(I) (2.0 mg, 13.9 µmol) was added to the solution and the solution was stirred for 72 h at room temperature. Then the solution was passed via SiliaMetS triamine to remove copper. The solvent was evaporated and the residue was purified by gel-filtration chromatography on Sephadex LH-20 eluted with MeOH.

The obtained solid was a white powder (47.5 mg, 6.59µmol, 83%). $Đ_M$(SEC)=1.06. MALDI–TOF–MS (matrix: CHCA) *m*/*z*: $[M+Na]^+$ calcd. for $C_{302}H_{497}N_{91}O_{113}Na^+$, 7232.59, found: 7232.59.

Supplementary Figures

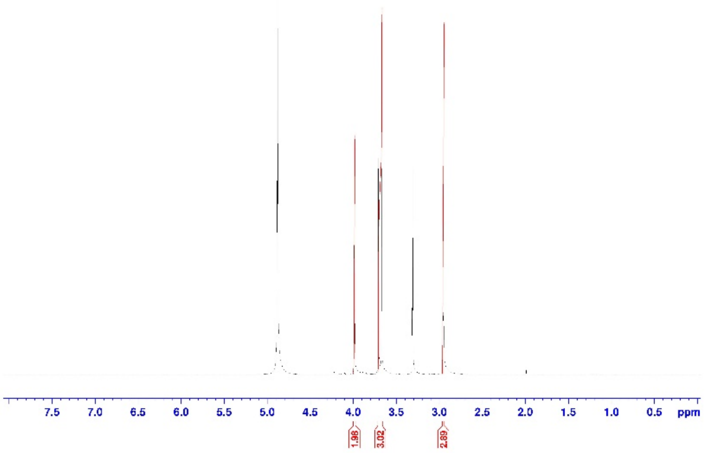

Figure S1. ^{1}H NMR spectrum of compound [**4**] in methanol-d_4. X-dimension is the chemical shift and red values are relative integral values.

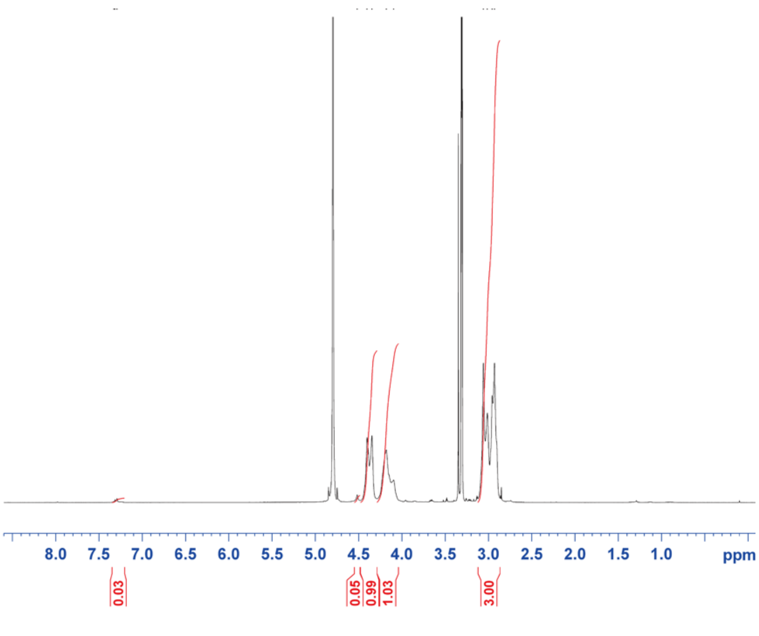

Figure S2. ^{1}H NMR spectrum of polymer [**1**] in methanol-d_4. X-dimension is the chemical shift and red values are relative integral values.

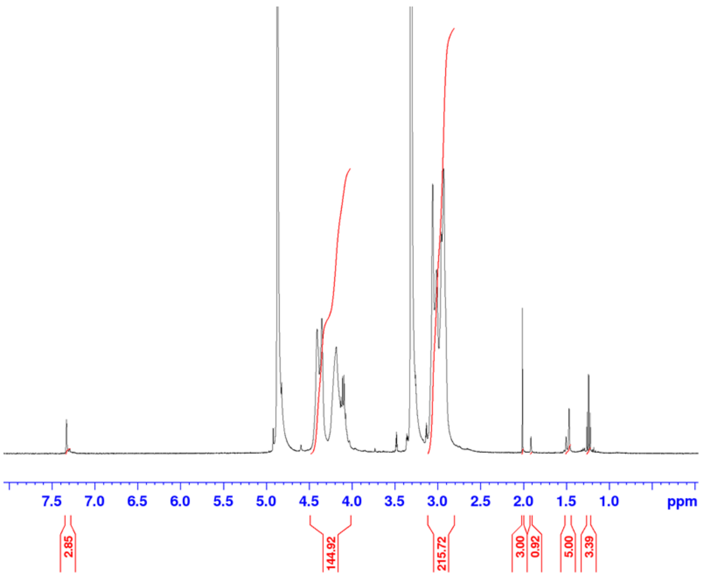

Figure S2. ^{1}H NMR spectrum of polymer **[6]** in methanol-*d_4*. X-dimension is the chemical shift and red values are integral results.

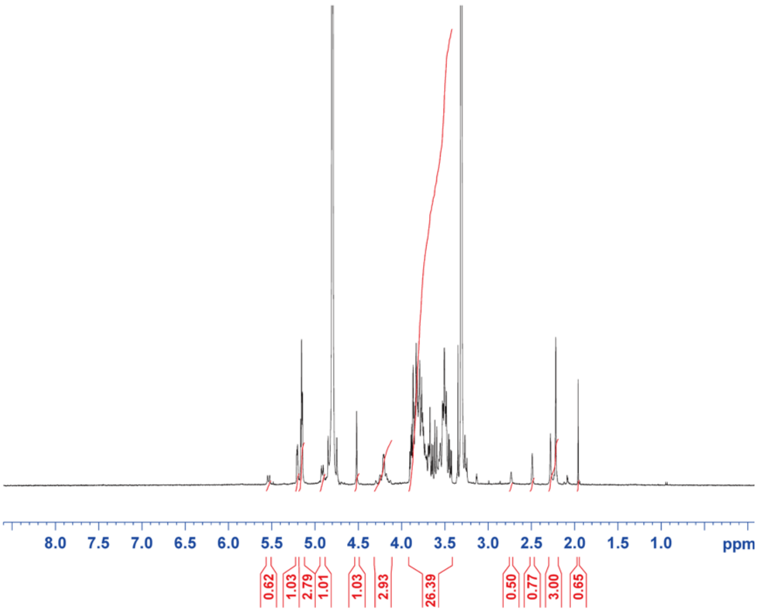

Figure S2. ^{1}H NMR spectrum of compound **[8]** in methanol-*d_4*. X-dimension is the chemical shift and red values are integral results.

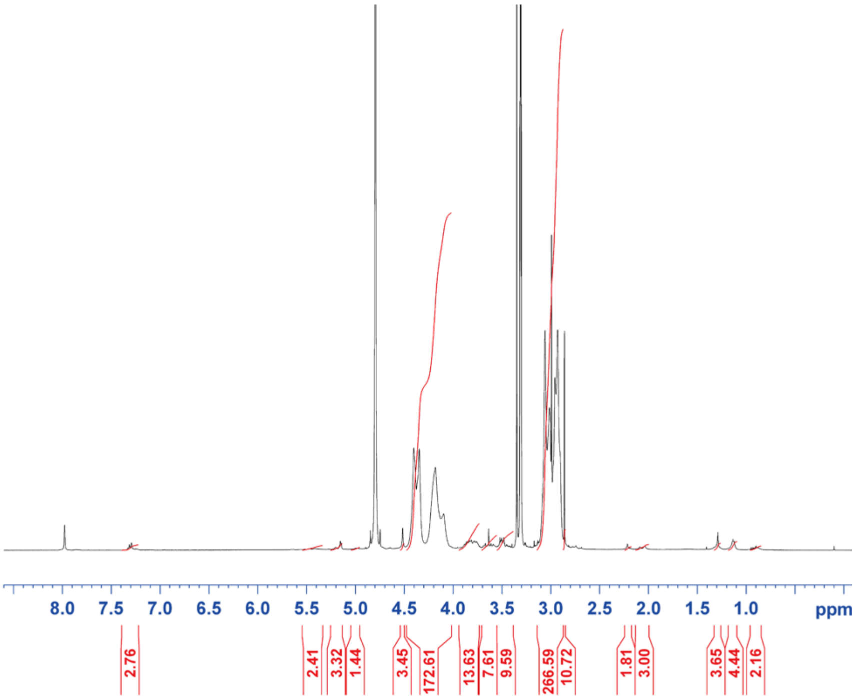

Figure S3. ^{1}H NMR spectrum of polymer **[2]** in methanol-d_4. X-dimension is the chemical shift and red values are integral results.

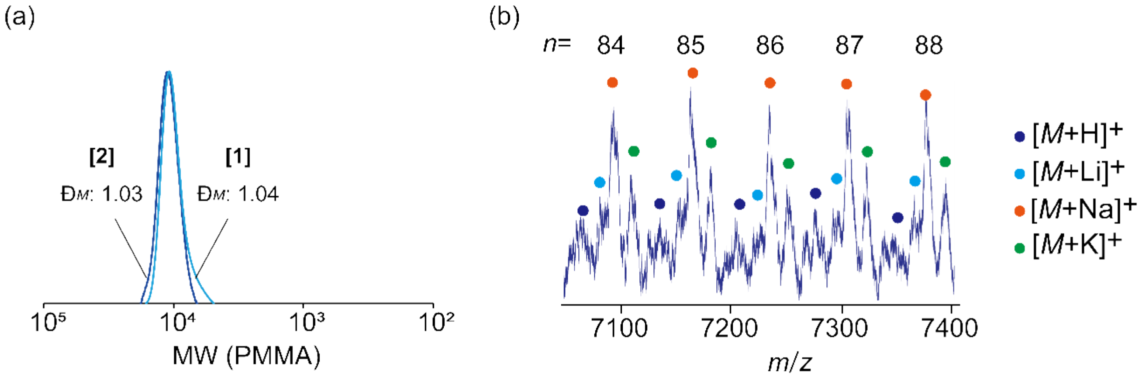

Figure S4. (a) Size-exclusion chromatograms of compounds **[1]** and **[2]**, in DMF containing 10 mM LiBr eluent). (b) MALDI–TOF–MS data of maltopentaose-*b*-poly(sarcosine)$_{86}$ (α-cyano-4-hydroxycinnamic acid used as a matrix agent).

Table S1. Size and its distribution of glycopeptoid self-assembly with different time of sonication by the bath type sonicator (40kHz, 130W) determined by DLS at 20 °C (*n*=5).

Sonication Time [min]	Polymer Conc.	Temperature [°C]	Z-average [nm]	PDI	Count Rate [Mcps]
1	10.0	20	148.6 ± 2.6	0.196 ± 0.012	18.7 ± 0.5
5	10.0	20	149.9 ± 2.8	0.181 ± 0.024	18.2 ± 0.4
10	10.0	20	149.6 ± 2.1	0.192 ± 0.020	17.8 ± 0.3

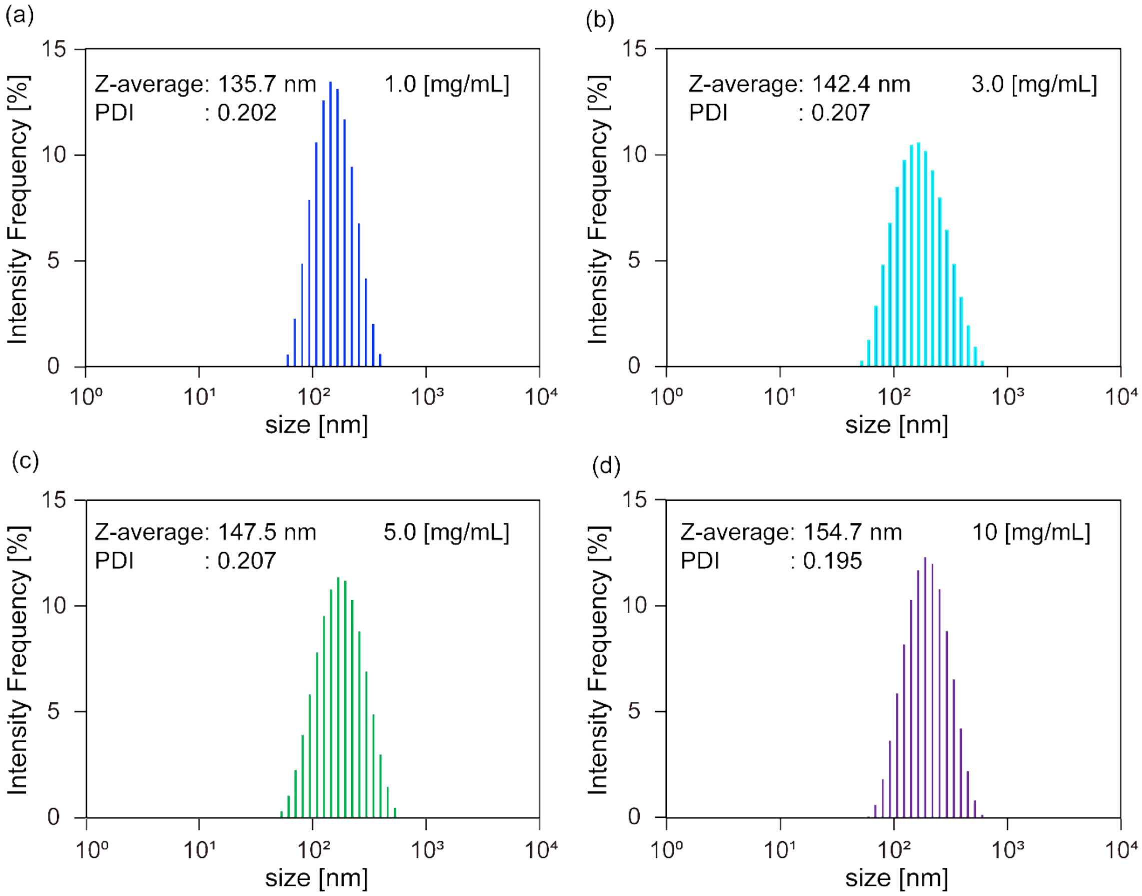

Figure S4. Dynamic light scattering profile of the self-assemblies formed by the glycopeptoid with different concentrations in water. All the glycopeptoid solution was sonicated for 1 min by the bath type sonicator (40kHz, 130W) at 20 °C and the DLS measurements were performed at 20 °C.

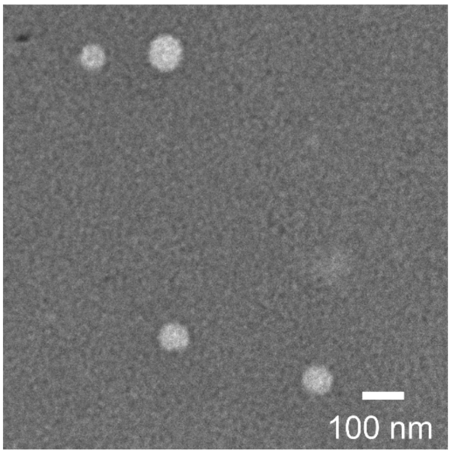

Figure S5. The TEM image of the compound **[2]** solution with negative staining using molybdenum ammonium (1.0 mg mL^{-1} in water).

Table S2. Size and the distribution of the glycopeptoid self-assembly with different temperature determined by DLS (*n*=5). Sonication was executed for 1 min by the bath type sonicator (40 kHz, 130W). The incubation time was 30 min at each temperature when the temperature was changed.

Polymer Conc.	Temperature [°C]	Z-average [nm]	PDI	Count Rate [Mcps]
10.0	10	149.6 ± 1.6	0.197 ± 0.009	18.1 ± 0.3
10.0	20	154.7 ± 1.2	0.195 ± 0.007	18.7 ± 0.5
10.0	30	157.5 ± 2.0	0.206 ± 0.011	17.8 ± 0.2
10.0	40	158.5 ± 1.0	0.202 ± 0.012	17.8 ± 0.1

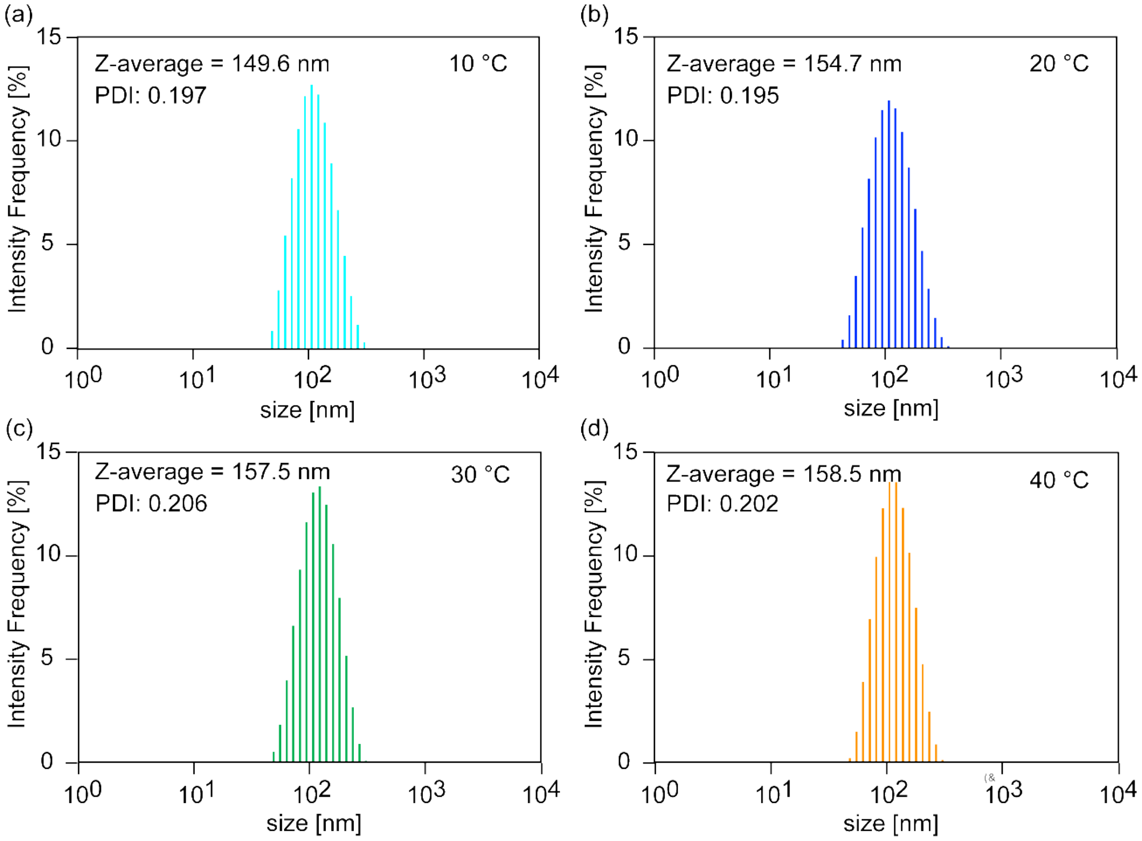

Figure S6. Dynamic light scattering profiles of the self-assembly with different temperatures in water. The glycopeptoid solution was sonicated for 1 min by the bath type sonicator (40kHz, 130W) at 20 °C and the DLS measurements were performed at 20 °C.

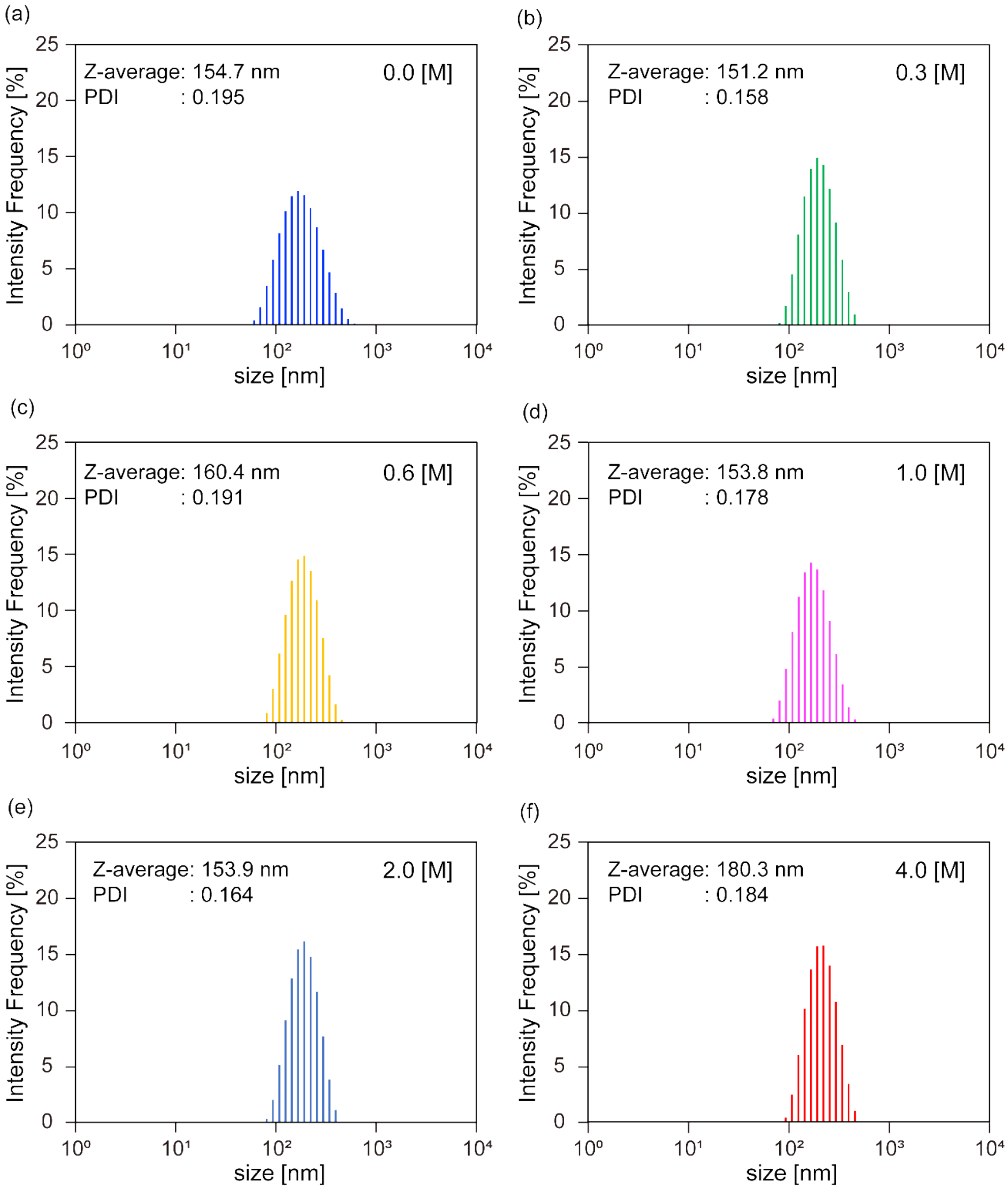

Figure S7. Dynamic light scattering profiles of the self-assemblies in different concentrations of the NaCl aqueous solution at 20 °C (10.0 mg/mL). All the glycopeptoid solutions were sonicated for 1 min by the bath type sonicator (40kHz, 130W) at 20 °C and the DLS measurements were performed at 20 °C.

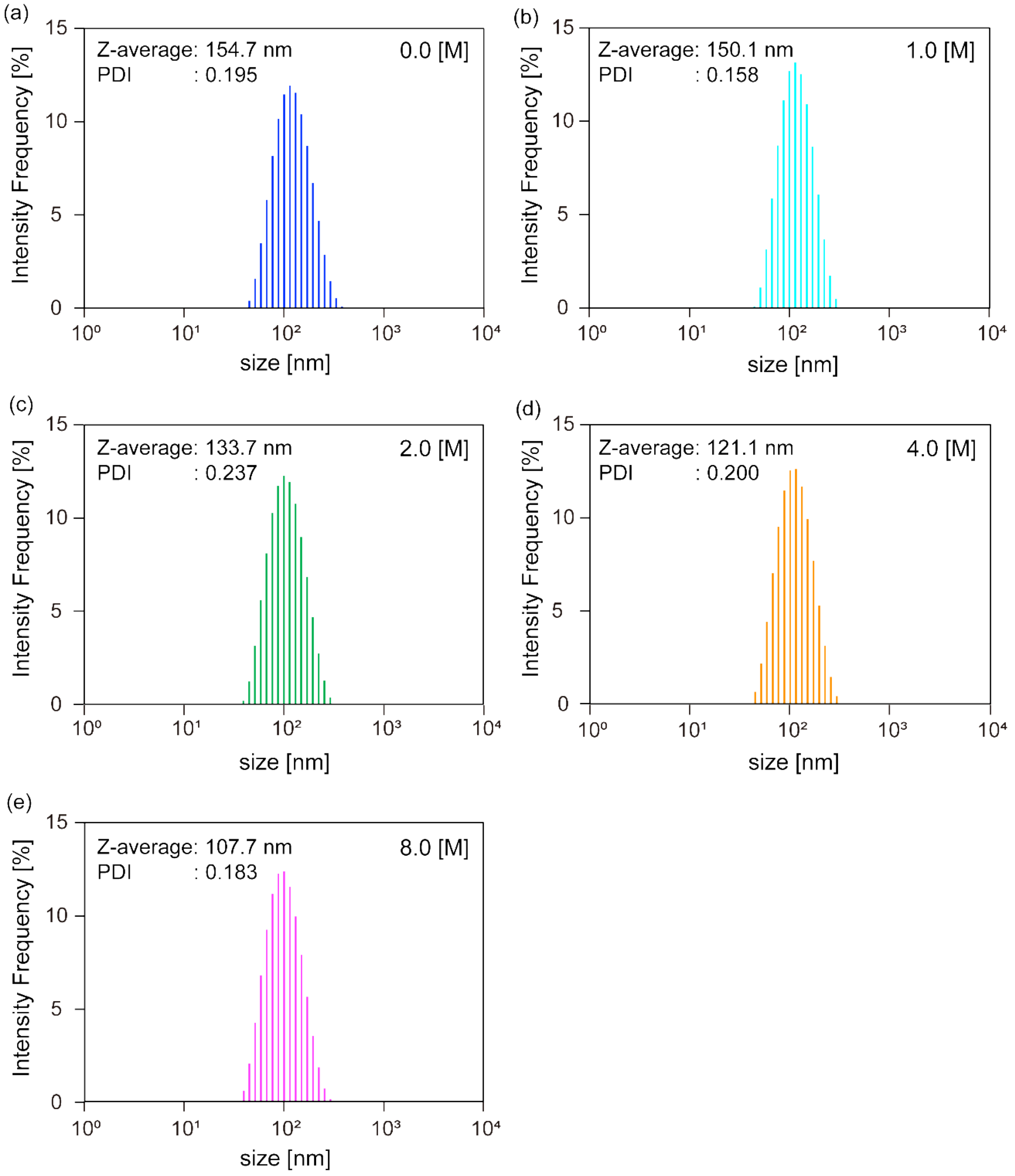

Figure S8. Dynamic light scattering profiles of the self-assemblies in different concentrations of the urea aqueous solutions at 20 °C (10.0 mg/mL). All the glycopeptoid solutions were sonicated for 1 min by the bath type sonicator (40kHz, 130W) at 20 °C and the DLS measurements were performed at 20 °C.

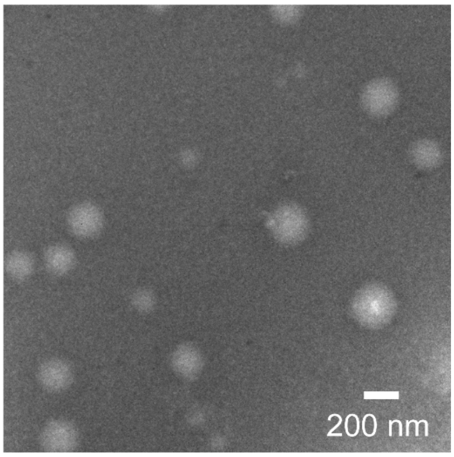

Figure S9. The TEM image of the compound **[2]** solution with negative staining using molybdenum ammonium in the 4 M NaCl solution (1.0 mg mL^{-1}). The glycopeptoid solution was sonicated for 1 min by the bath type sonicator (40kHz, 130W) at 20 °C.

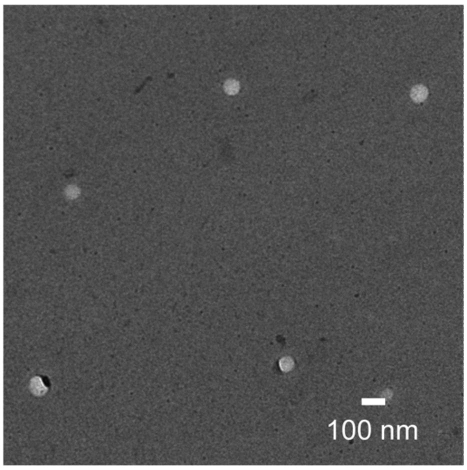

Figure S10. The TEM image of the compound **[2]** solution with negative staining using molybdenum ammonium in the 8 M urea solution (1.0 mg mL^{-1}). The glycopeptoid solution was sonicated for 1 min by the bath type sonicator (40kHz, 130W) at 20 °C.

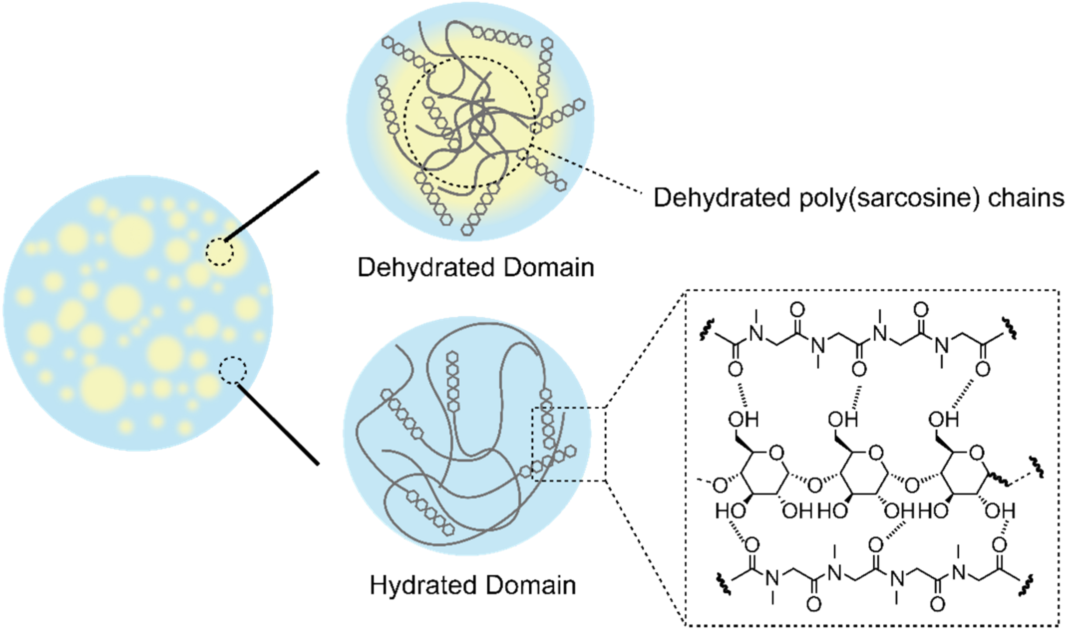

Figure S11. The schematic representation of the structure of the coacervate of the glycopeptoid.
